# Association of Drug Application and Hydration Status in Elderly Patients

**DOI:** 10.3390/nu13061929

**Published:** 2021-06-04

**Authors:** Laura Hoen, Daniel Pfeffer, Rico Zapf, Andrea Raabe, Janosch Hildebrand, Johannes Kraft, Stefan Kalkhof

**Affiliations:** 1 Institute for Bioanalysis, Coburg University of Applied Sciences and Arts, Friedrich-Streib-Str. 2, D-96450 Coburg, Germany; Laura.Hoen@hs-coburg.de (L.H.); Daniel.Pfeffer@stud.hs-coburg.de (D.P.); Rico.Zapf@stud.hs-coburg.de (R.Z.); Janosch.Hildebrand@hs-coburg.de (J.H.); 2 Division of Geriatrics, Klinikum Coburg GmbH, Ketschendorfer Str. 33, D-96450 Coburg, Germany; Johannes.Kraft@Klinikum-Coburg.de; 3 Division of Nephrology, Klinikum Coburg GmbH, Ketschendorfer Str. 33, D-96450 Coburg, Germany; Andrea.Raabe@gmx.de; 4 Protein Biomarker Unit, Fraunhofer Institute for Cell Therapy and Immunology, Perlickstr. 1, D-04103 Leipzig, Germany

**Keywords:** drug-nutrient interactions, nutrition, body composition, geriatrics, nutrition assessment, malnutrition, clinical nutrition, prevention

## Abstract

Due to multifactorial reasons, such as decreased thirst and decreased total body water, elderly patients are vulnerable to dehydration. Mild cognitive impairment (MCI) or dementia increase the risk of dehydration and, in turn, dehydration decreases cognitive performance. The study aims to identify and assess differences in hydration status, taking into account patients’ drug treatment and diseases, using bioelectrical impedance vector analysis (BIVA), thereby revealing unfavorable aspects of prognosis. 447 geriatric patients (241 women, 206 men) including information on medication and bioelectrical impedance analysis (BIA) were investigated, which allowed studying the association between 40 drugs and the hydration status. First, patients were divided into disease groups. Renal disease and diuretic treatment were significantly different in both sexes, whereas cardiovascular patients differed exclusively for females. Next, drug enrichment was examined in either hyperhydrated or dehydrated patients. Simvastatin, candesartan, bisoprolol, amlodipine, olmesartan, furosemide, torasemide, allopurinol, mirtazapine, pantoprazole, cholecalciferol, and resveratrol showed enrichment depending on hydration status. This study demonstrated that patients can be differentiated and stratified by BIVA, taking into account medication and disease associated with hydration status. Although patients diagnosed with MCI and therefore treated with resveratrol, BIVA still showed evaluated dehydration. This is unfavorable in terms of prognosis and requires special attention.

## 1. Introduction

The demographic change leads more and more to an older population (age > 65) [1]. Due to multifactorial reasons, like reduced thirst, smaller fluid reserve and decreased total body water, this population is susceptible to dehydration and electrolyte abnormalities [2]. In addition to physical changes and inadequate fluid intake, different age-associated medication leads to a higher dehydration risk [3]. A variety of drug-induced mechanisms like diarrhea, increase of urine volume, decrease of thirst sensation, increase of sweat production or decrease of appetite alter the hydration status [4]. Diuretics, e.g., increase the urine volume, which is indicated to treat edema, but unmonitored use can result in dehydration [3,5]. Furthermore, mild cognitive impairment (MCI) or dementia result in a higher dehydration risk [2,6,7], and in return dehydration decreases the cognitive performance [8,9]. Woiszel [10] recently showed that there is a trend for higher odds for impending dehydration when patients take procognitive medications and the negative effect of this becomes significant when adjusting for dementia. The symptoms of dehydration have a huge range between simple dizziness, confusion to seizures and deaths [2]. Nevertheless, diagnosing dehydration is very challenging because diagnostic signs like capillary refill time, abnormal skin turgor or respiratory pattern, which are widely used in children, cannot be applied to the elderly because of the aging process [2]. The bioelectrical impedance analysis (BIA) is thereby a relatively inexpensive, easy-to-use and non-invasive method for measuring the body composition [11]. By applying alternating electrical current the bioelectrical impedance analyzers determine the two components of bioelectrical impedance (Z) in the human body; the resistance (R) and reactance (Xc) [12]. The bioelectrical impedance vector analysis (BIVA) uses these individual components, normalized by height (H) of the subject, and represents them in the RXc graph (abscissa, R/H; ordinate, Xc/H) [13]. As reactance is directly related to the amount of soft tissue structures and resistance is inversely related to the intra- and extracellular water [14]; BIVA is a very promising tool for assessing a patient’s hydration and nutritional status [7,12,15]. Besides to the impedance measurement error the biological variability of subjects like age, sex, race, and body mass index (BMI) affects this approach [7,16].

This study aimed to show differences in patient’s hydration status considering their medicament treatment or diseases by BIVA and thereby reveal unfavorable aspects of prognosis.

## 2. Material & Methods

### 2.1. Study Design

In 2017, 2018, and 2019, 471 outpatients treated in the Department of Geriatrics of Prof. Dr. Kraft (Regiomed Klinikum Coburg) were studied retrospectively. All patient data were included in this study in a blinded fashion and will be referred to as the Coburg cohort in the following. Inclusion criteria for the patients were age (between 65 and 98 years old (79.8 ± 7.0 (SD)) and a bioelectrical impedance analysis received at least once. The following data were collected at the date of the clinical investigation: BIA measurements, laboratory values (hematocrit, urea, sodium, creatinine and glucose), Clock-Drawing Test, Mini-Mental State Examination and medication.

Preparation of the patients for BIVA was according to their medication and treatment plan. Because of the retrospectivity physical activity, dietary or any other nutritional aspects than medication, which may also affected the result of the BIVA analyses, were not included in this study.

### 2.2. Bioelectrical Impedance Vector Analyses

BIA examinations were performed as part of routine inspections of geriatric patients who were not conditioned in the course of dieting, exercise, etc.

Body composition was assessed with an InBody 770 multi-frequency (1, 5, 50, 250, 500, 1000 kHz), four-point bioelectrical impedance device (InBody 770, Cerritos, CA, USA). On this basis, the parameters phase angle, total body water (TBW) and extracellular water (ECW) were subsequently calculated. Impedance and reactance values were utilized to calculate Bioelectrical Impedance Vector Analyzes (BIVA) values as been described by Piccoli et al. [17]. For the analysis the resistance is needed, therefore the following formula is used: Z^2^ = R^2^ + Xc^2^; R, Z and Xc values of the whole-body were calculated as the sum of those readings for the right arm, right leg and trunk at 50 kHz measured with an electric current intensity of 80 µA [18]. BIVA analyses are done with the formulas provided by Piccoli [19]. To investigate the population distribution, the BIVA values of the patients were plotted against the reference populations in the BIVA Software [19]. As BIA is influenced by sex [16], males and females were compared separately. Sex-specific age and BMI matched population of the study of Roubenoff et al. [20] were used as reference.

The Z-Score is generally calculated by dividing each; resistance and reactance, with the height, following the transformation by mean and standard deviation of a suitable reference population. Based on the Coburg patient’s data a new reference population was generated considering sex, age and body mass index (BMI). This generated reference population was used for building the Z-Score Ellipse to classify the 447 patients (see Section 2.4).

### 2.3. BIA Confidence Intervals of Cardiovascular, Renal, Diuretic or Dementia Patients

The patients were divided into subgroups: cardiovascular, renal, diuretic or dementia according to their medication. All groups were chosen because of their relation with the hydration status and their increased occurrence (see Section 3.1). The following medications and laboratory values were used to divide the patients into the corresponding groups: *Cardiovascular* = bisoprolol, nebivolol, metoprolol, carvedilol, candesartan, ramipril, lisinopril, enalapril, atorvastatin, ezetimibe/atorvastatin, simvastatin, amlodipine; *Renal* = allopurinol and an elevated level of creatinine (>97 µmol/L (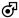)/>80 µmol/L (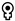)); *Diuretic* = torasemide, furosemide, eplerenone, spironolactone, chlortalidone, hydrochlorothiazide (HCT); *Dementia* = memantine, donepezil, Ginkgo biloba extract, rivastigmine, striking Mini-Mental State Examination (MMSE) (Score ≤ 23) and Clock-Drawing Test (Score ≥ 3). As reference groups, all patients were applied, who were not been classified for the specific disease. Using this classification system 89% of all patients (397 of 447) were included in at least one of the groups. However, group assignments were not disjunctive, e.g., an individual which was included in the cardiovascular group can also additionally be listed in the renal group (Supplementary Figure S1). The groups were split up by sex. For each patient subgroup the mean, standard deviation and linear correlation coefficient between the components resistance/height and reactance/height were calculated. Differences between the mean impedance vectors in the described groups were assessed with Hotelling’s T^2^ test and graphically with 95% probability confidence ellipses, which correspond to a statistically significant difference between mean vector displacements on the RXc graph (*p* < 0.05). A significant Hotelling’s T^2^ test is equivalent to a significant difference in R, Xc or both parameters.

### 2.4. Classification of Patients According to the Hydration Status and Enrichment of Medicaments

**Figure 1:**
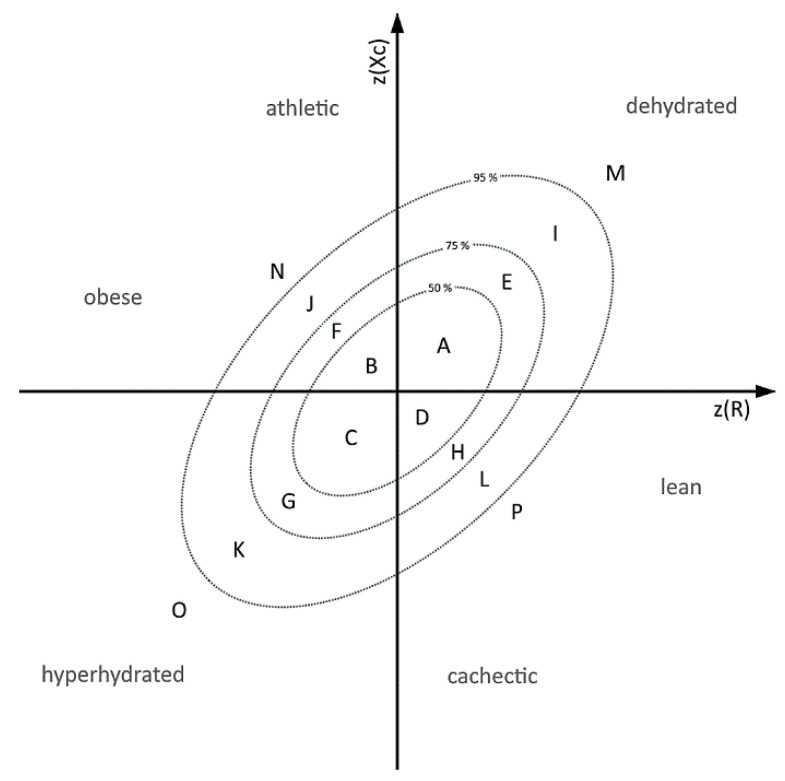
Subdivision Model of Z-Scores based on 50%, 75% and 95% tolerance ellipses A: Annotation of the 50%, 75% and 95% tolerance ellipses based on Norman et al. [21]. Sectors M and I (dehydrated), K and O (hydrated), and A, B, C, D (control) were defined to classify the patients according to the hydration status.

To have a look on individual medication enriched in different hydration statuses, a Z-Score model based on Norman et al. [21] was outlined and the patients were divided into three groups, based on the patient’s position in this Z-Score model (Figure 1) A: (i) dehydrated patients located in sector I and M; (ii) normally hydrated located in sector A, B, C and D; (iii) hyperhydrated located in sector O and K. All patients appearing in the other sectors (E, G, F, H, J, N L, P) were excluded from the following analysis. The absolute frequencies of each prescribed drug and the number of patients in each group were computed. Based on these values the relative frequencies within these groups were calculated. The fold change (FC) was defined as the ratio of relative frequency in one of the extreme groups (hyperhydrated or dehydrated) and the median of the relative frequencies of the remaining other two groups (normal hydrated plus the remaining extreme group). Enriched drugs were defined by an occurrence in the population study ≥ 15 for statistical relevance and a binary logarithm of log_2_(FC) ≥ |0.5| as minimal difference between the groups and a significant difference in the RXc graph (Hotelling’s T^2^ test) in minimum one gender. The significance was checked by Fisher’s exact test in which each extreme group was compared to the normal hydrated group. Using the Benjamini & Hochberg’s method *p*-values were adjusted for multiple testing.

## 3. Results

### 3.1. Coburg Patients Are Leaner and Hyperhydrated in Comparison to Reference Population

**Figure 2:**
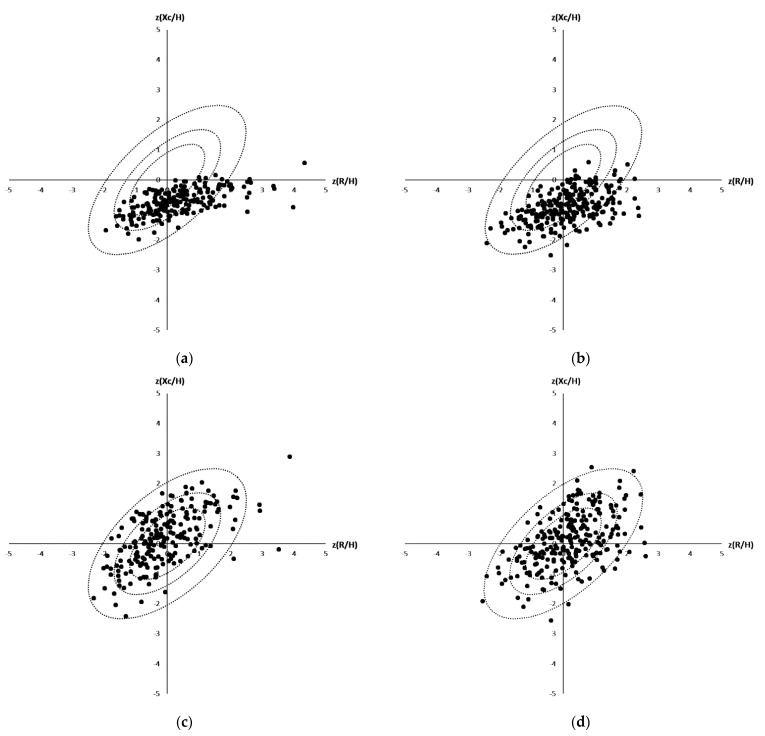
(**a**–**d**): Distribution of the Coburg patients in a Bioelectrical Impedance Vector Analyses (male (**a**,**c**), female (**b**,**d**)) plotted against reported tolerance intervals of the reference population reported by Roubenoff et al. (**a** + **b**) and against tolerance ellipses calculated based on all 447 patients being included in the Coburg’s cohort (**c** + **d**).

BIA analyses of 471 outpatients were performed in 2017 to 2019 in the geriatric clinic in Coburg. Twenty-four of these contained incomplete patient information and were therefore excluded, while the remaining 447 (241 women, 206 men) were used for further analysis. The average age of the 447 outpatients, included in this study (called Coburg cohort), was 79.8 ± 7.0 years, and 54% (241) of them were female. Main diagnoses included cardiovascular disease (61%, 277), dementia (53%, 237), renal disease (33%, 149), and diuretic treatment (33%, 149). Initially, differences between the study of Roubenoff et al. [20], which included 455 elderly patients (161 men average age of 78.2 ± 4.3 years and 294 women 78.4 ± 4.5 years, 99% of whom were White) being acquired in the context of the Framingham Heart Study (USA), were discovered (Figure 2a,b). The used reference populations, which build the tolerance ellipses, were chosen because they matched most in terms of age, BMI and sex. Plotting the BIA data of the Coburg patients in the normalized tolerance ellipses published by Roubenoff et al. [20] revealed a shift to lower Z(Xc/H) (male: −0.72 ± 0.43, female: −0.88 ± 0.43) and higher (R/H) (male: 0.31 ± 1.07, female: 0.14 ± 0.93) values. According to Piccoli et al. [17] this means that the Coburg patients can be classified to be more hydrated, cachectic and lean because they were laying predominantly in the lower right part. To take these shifts between the Coburg and the Roubenoff et al. [20] cohort into account and to investigate differences within the Coburg group, a new reference population out of the Coburg people was build and the required reference values were calculated. The BMI for male and female population is between 25 and 28 kg/m^2^ and an age between 65 and 98. Male population defined by *n* = 130 (number), m_R_ = 290.7 (R/H mean), s_R_ = 35.8 (R/H standard deviation), m_Xc_ = 21.4 (Xc/H mean), s_Xc_ = 4.4 (Xc/H standard deviation), r = 0.5740 (Correlation (R/H, Xc/H)). The female population is defined by *n* = 115, m_R_ = 360.6, s_R_ = 42.6, m_Xc_ = 25.1, s_Xc_ = 4.9, r = 0.6061. With these new build reference populations, the 447 patients were located in the Z-Score Ellipses and as expected, nearly 50% (225 of 447) were lying in the middle in the sectors A, B, C and D (Figure 2c,d).

### 3.2. Differences in the Group of Cardiovascular, Dementia, Renal and Diuretic Patients

**Figure 3:**
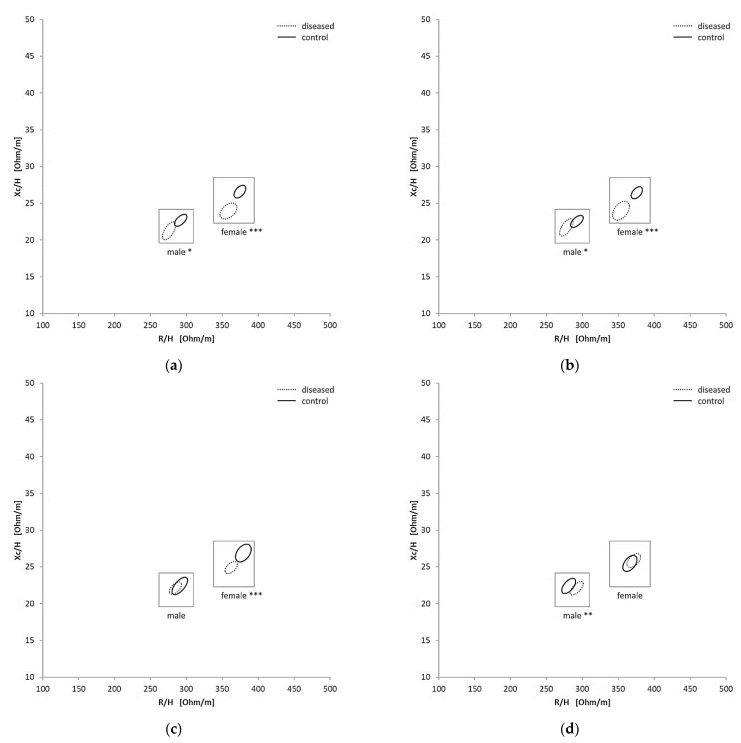
Confidence Ellipses in the RXc graphs for the different subgroups and each gender: renal (**a**), diuretic (**b**), cardiovascular (**c**) and dementia (**d**), * = *p* < 0.01, ** = *p* < 0.005, *** = *p* < 0.005.

To investigate how the BIVA values were altered due to the presence of diseases, patients were divided into four groups (cardiovascular, dementia, diuretics and renal). There was a significant variation in the RXc graph for female and male between the renal disease (*p* 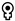 < 0.0001; *p* 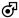 = 0.0274) and their control group and between patients under diuretic treatment (*p* 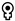 < 0.0001; *p* 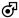 = 0.0106) and their control group (Figure 3a,b). Comparing the patients with cardiovascular disease and those without cardiovascular disease, there was a significant difference in the female group (*p* < 0.0001), but not in the male group (*p* = 0.5452) (Figure 3c). The dementia patients could be significantly discriminated in the male group (*p* = 0.0074) (Figure 3d), but not in the female group (*p* = 0.6145). Because a clear differentiation between the subgroups could be seen, the enrichment of drugs in extreme groups (hyperhydrated and dehydrated) was subsequently investigated.

### 3.3. Enrichment of Medication According to Patient’s Hydration Status

However, due to the obvious difference between the Coburg patients and the provided reference, a generation of a reference population out of the Coburg patients to improve the categorization of the patients was necessary. For the Coburg cohort, the reference values were mentioned in Section 3.2. Thereby the patients could be divided into dehydrated, normal hydrated and hyperhydrated according to their position in the vector graph. For each group, the baseline characteristics and the main impedance values were shown in Table 1. The ratio between male and female was nearly equal in all of the three groups. The hyperhydrated group was smaller than the dehydrated group. The biggest group was the normal hydrated group, in the defined sectors (A, B, C and D) and should include ca. 50% of all patients (here 225 out of 447, Figure 2c,d). Dehydrated patients had a lower BMI (24.1 ± 3.6) in comparison to the hyperhydrated patients (28.6 ± 4.8) (*p* = 0.0001), whereas the Sodium level was not significantly different (*p* = 0.1315). Dehydrated and hyperhydrated patients were slightly younger than the normal hydrated group (*p* < 0.05). As expected dehydrated patients had higher impedance, reactance and resistance values than the normal hydrated group. All bioelectrical impedance values were significantly different between these groups (*p* < 0.005). It was striking that the creatinine level was lower in the dehydrated group in contrast to the normal hydrated (*p* = 0.002) and hyperhydrated group (*p* = 0.0002).

Using the different hydration subgroups, the enrichment of specific medication was investigated. An enrichment of different drugs in both extreme groups of dehydrated and hyperhydrated patients could be seen with a higher enrichment in the hyperhydrated group (Table 2).

**Figure 4:**
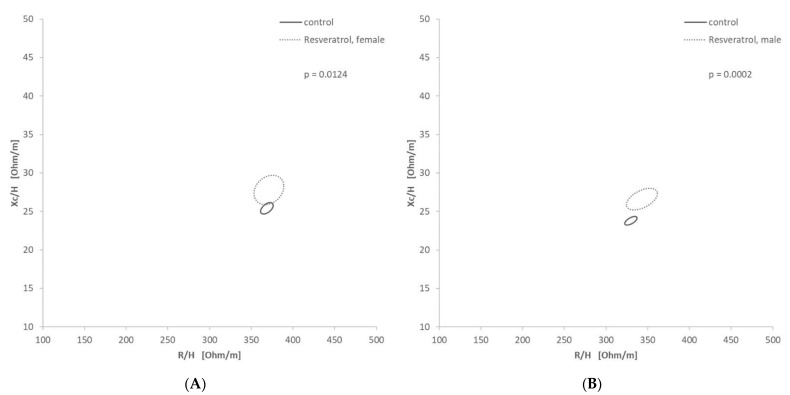
Confidence Ellipses in the RXc graph for resveratrol: (**A**—female, **B**—male) Significant differentiation of male (*p* = 0.0158) and female (*p* = 0.0124) patients receiving resveratrol.

According to their indication, drugs were classified into the groups (i) cardiac, (ii) diuretic, (iii) endocrinologic, (iv) mild cognitive impairment, (v) psychotic/neurological, (vi) gastrointestinal, and/or (vii) nutritional supplements, to get an overview of the enriched indications. In total of the 40 drugs given to at least 15 patients, 12 drugs were found to be significantly (log_2_(FC) > 0.5, *p* < 0.05 in minimum one gender) more prescribed in hyperhydrated patients, whereas five were more consumed by dehydrated patients compared to normal hydrated patients (Table 2). Especially cardiac associated medication like candesartan and amlodipine, was enriched in the hyperhydrated group. This underlines the results showed before (see Section 3.2). There was one gastrointestinal medication, pantoprazole, enriched. Allopurinol, a renal medication, was enriched in the hyperhydrated group. In the hyperhydrated group also two diuretics were enriched, torasemide and furosemide. Furosemide with a 12-times higher enrichment. Furthermore, Olmesartan and amlodipine/olmesartan were found to be enriched. In both extreme groups, cardiac associated medication was the most common representative (54% of the enriched medication of each group). In the dehydrated group mirtazapine, an antidepressant, and resveratrol, a nutritional supplement used for MCI treatment, could be found more often. The RXc-graph of Resveratrol (Figure 4) showed for both genders lower Xc values and minimal lower R values in the control group, which is not receiving resveratrol.

## 4. Discussion

### 4.1. Diseases Associated with Hydration Status Are Differentiable via Bioelectrical Impedance Analyses

BIA is an extensively used, noninvasive method to assess the body composition, which allows evaluating important body compartments such as fat mass, intra- and extracellular water content or the overall hydration status [11]. Furthermore, BIA has been reported to indicate the presence of diseases such as catabolism in Type 2 diabetes [22], gastrointestinal disease [23], Alzheimer/mild cognitive impairment [24], edema [25] and stress/inflammatory biomarkers [26]. The multifrequency device used provides data regarding intracellular and extracellular water content as well as phase angle. Based on these values, hydration classification can also be performed. However, compared with BIVA, a lower association was observed between hydration status, defined by these values, and drug or disease enrichment (data not shown). Therefore, this study focused on the association of hydration classification determined by the BIVA method. However, due to the fact that not only sex and BMI alter the BIVA assessment but also the ethnics [16], a new reference population of the Coburg cohort was defined. This was done to get a proper classification of the patients into the shown groups of normal hydrated, dehydrated and hyperhydrated patients and to redefine classification within the BIVA ellipses.

For all of the four main categories (renal, cardiovascular diseases, diuretics and dementia) alteration of fluid status of a patient could be expected. Nevertheless, only renal disease and diuretic treatment were significantly differentiated in both genders. Diuretics increase the urine volume in order to treat e.g., edema [5] and thus affect the hydration status. It has been shown that bioelectrical impedance values are significantly decreased when edema is on the body side which is measured [27]. The group of patients with renal disease was defined over an elevated level of creatinine and the intake of Allopurinol. Allopurinol, a xanthine oxidase inhibitor, is used to treat elevated levels of uric acid in the blood [28]. There is a complex relationship between serum urate and kidney function [28,29]. Dehydration leads to increased reabsorption of water in the kidneys [2]. Thereby urea is also reabsorbed. Elevated levels of creatinine, a product of muscle metabolism excreted by the kidneys, are associated with acute kidney injury [30,31]. The kidney regulates body fluid volume [32] and thereby alters the hydration status. The patients group of cardiovascular vs. non-cardiovascular patients could be discriminated in the female group, however, for the males the differentiation did not reach significance. The hydration status influences vascular function and cardiovascular regulation. Dehydration impairs cutaneous vascular function and alters blood pressure regulation at rest, during exercise and orthostatic stress [33]. Also in combination with orthostatic dysregulation, dehydration leads to a higher risk of falling, which is an important risk factor for morbidity and mortality in geriatric patients [34]. On the other hand, hyperhydration causes an increased cardiovascular preload and blood pressure [35], which leads to a higher risk for heart failure and stroke [36]. Although the dehydration risk is known to increase with dementia [6], the dementia group only differed significantly in the male group (Figure 3d). The smaller group size of the female patients may was a reason for this.

### 4.2. Several Medications Are Enriched in Hyperhydrated and Dehydrated Patients

However, even if possible effects of polypharmacy, sociodemographic or dietary characteristics of patients were not taken into account or were corrected for, 12 medications were found to be significantly more frequently present in dehydrated and/or hyperhydrated patients. Furthermore, there were more enriched drugs in hyperhydrated patients (eight drugs) compared to the dehydrated patients (four drugs) (Table 2). In comparison to the Clinical Dehydration Score BIA tends towards values indicating Hyperhydration [37]. Since the hyperhydrated group of the study was smaller than the dehydrated group, this aspect should not have any influence on the shown data. The largest class of active ingredients was used in cardiovascular diseases in which excessive water retention, e.g., formation of edema, is typical [38]. Interestingly, two diuretics were enriched in the hyperhydrated group; even though their indications are the treatment of e.g., fluid build-ups. The absence of patients in the dehydrated group showed that the patients are not over treated. Also, Ohara [39] showed that furosemide reduces extracellular water but not intracellular water, which leads to a shift of extracellular water to total body water. Coodley [40] noticed a statistic relevant elevation of resistance and reactance due to furosemide intake as the equitation used for BIA seems not valid in people with congestive heart failure. This finding can explain the elevated level of furosemide in the hyperhydrated group as these values form the base of the calculations. Torasemide, also a loop diuretic like furosemide, may have the same effects but there is no research yet and further analysis is needed to verify this thesis. On the other hand, hyperhydration is the requirement for initiating a diuretic treatment, so maybe there is low treatment response or only moderate dosing to avoid dehydration in this valuable geriatric patients cohort measured with the BIA. In the dehydrated group olmesartan, an angiotensin receptor blocker, used for the treatment of high blood pressure [41], is enriched. It has the potential for inducing dehydration. Marietta [42] showed that this drug-induced diarrhea which also can lead to chronic diarrhea and thereby common complications like dehydration and acute kidney injury occur [42]. Also olmesartan, especially in combination with amlodipine, can cause diuresis [43]. As olmesartan was enriched in the dehydrated group, BIVA may be used as an early warning. Mirtazapine seemed to be not indicated of being found in the dehydrated group. It is usually used as an antidepressant but according to Prof. Kraft, his patients received it as an appetizer because increased appetite is one of the most common side effects of this drug [44]. But it is also known that Mirtazapine decreases the thirst sensation [45], which may be an additional explanation for the finding of this drug in the dehydrated group. The discussed effects of the individual drugs have no direct causality. So it is possible, but not necessary, that olmesartan, for example, causes diuresis.

### 4.3. Dehydration and Mild Cognitive Impairment Can Lead to Mutual Deterioration

Bickel et al. [46] showed that in patients with dementia, dehydration and electrolyte imbalances, as well as urinary tract infections and lower respiratory tract infections, were more common. Interestingly, resveratrol is found in the dehydrated group. In a dose of 250 mg per day, it was given to improve mild cognitive impairment. MCI is defined as a syndrome of cognitive decline. It is a stage between normal aging and the more serious state of dementia [47]. Different studies showed that resveratrol improves mild cognitive impairment [48,49], which should have a positive influence on the hydration state. But the patients are still dehydrated according to their BIVA values. Additionally, dehydration decreases cognitive performance [8,9], which can lead to a further deterioration in the cognitive state. This is implicating that patients diagnosed with MCI need closer controls of their hydration status. A geriatric study by Wojszel used 14 predictors including taking procognitive medication in a multivariable logistic regression analysis, which showed a trend in higher odds for impending dehydration for this predictor. It even becomes significant when dementia as a variable was added to procognitive medications. Both models showed an overall prediction success rate of 65.7% [10]. This emphasizes the results for Resveratrol in this study. Despite the increased dehydration risk, several epidemiologic studies reported an association between adiposity and developing Alzheimer or dementia [50,51]. But Camina et al. [52] showed that BIVA reflects dementia-related changes in body composition better than BIA and found that patients with dementia have lower Xc values. The RXc graph (Figure 4) revealed higher Xc values for patients receiving resveratrol. Therefore, the Xc value can be used as a diagnosing factor that should be regarded to minimize the risk for further cognitive decline.

### 4.4. Limitations

In contrast to the information on which drugs the patients were taking at the time of the study, there was only insufficient and, due to the still statistically too low number of patients, non-significant information on how long and with which comedication the patients had already taken the drugs. However, this could have a strong influence in part because, for example, thiazide diuretics may cause different acute and chronic reactions depending on the duration of use. Also, the possible effects of polypharmacy or sociodemographic or dietary characteristics of patients were not directly considered. However, this may well have a significant impact on hydration status, which is further indicated by the marked shift in Z-score models in the Coburg cohort compared with the American Framingham Heart Study [20] (see Section 4.1). Nevertheless, the aspect that the study was not decisively adjusted with respect to these effects and still showed significant associations underlines the relevance of the results. However, it should be considered in return that a correction of the Z-score model for an application to further cohorts should be checked and, if needed, also performed.

Furthermore, an association with hydration status was shown and discussed for several drugs (see Section 4.2). However, it must be kept in mind that direct causality cannot be inferred from the associations shown for the drugs.

## 5. Conclusions

This study showed that there are associations between the hydration status of elderly patients determined by bioelectrical impedance analysis and disorders such as cardiovascular, dementia, renal, and diuretic diseases. Furthermore specific medication, several not yet associated with the altered fluid status, were significantly enriched in either dehydrated or hyperhydrated patient groups.

Resveratrol (antidementive), olmesartan (antihypertensive), and mirtazapine (antidepressant) were more frequently prescribed in dehydrated patients. Beyond dehydrated patients also in hyperhydrated patients drugs, such as furosemide and torasemide (diuretics), simvastatin, candesartan, bisoprolol, amlodipine (cardiac medication), pantoprazole (proton pump inhibitor), cholecalciferol, (vitamin D supplement) and mirtazapine are enriched. Remarkably, patients diagnosed with even mild cognitive impairment who received resveratrol as supportive therapy in this study group showed reduced hydration levels despite intensive geriatric care. This underscores the need for more attention to this prognostically unfavorable aspect.

## Figures and Tables

**Table 1:** Baseline Characteristics and impedance values of the patients classified by hydration status (see also Figure 1), average ± standard deviation, * = *p* ≤ 0.05, ** = *p* ≤ 0.005, *** = *p* ≤ 0.0001.

Characteristics	Dehydrated	Normal-Hydrated	Hyperhydrated
N	46	225	27
Male/Female	18/28	109/116	13/14
Height [cm]	165.4 ± 9.5	167.7 ± 9.3	173.6 ± 8.9 **
Age [yr]	78.3 ± 8.5 *	80.6 ± 6.4	77.9 ± 6.6 *
Weight [kg]	66.1 ± 12.8 **	73.1 ± 11.8	86.1 ± 15.7 ***
BMI [kg/m^2^]	24.1 ± 3.6 **	26.0 ± 3.4	28.6 ± 4.8 **
Sodium [mmol/L]	141.7 ± 2.6	141.0 ± 2.9	141.6 ± 2.8
Hematocrit [L/L]	0.417 ± 0.036 **	0.398 ± 0.038	0.382 ± 0.050 *
Urea [mmol/L]	9.1 ± 8.2	7.7 ± 3.5	7.6 ± 0.9
Creatinine [µmol/L]	82.5 ± 28.8 **	99.2 ± 33.9	114.9 ± 42.1 *
Glucose [mmol/L]	5.9 ± 1.4 *	6.6 ± 2.2	6.3 ± 1.4
Total Number of Medication	5.2 ± 3.6 **	7.6 ± 4.3	9.3 ± 4.0
PhA [°]	4.5 ± 0.7 **	4.1 ± 0.4	3.8 ± 0.8 **
Z [Ohm]	647.8 ± 64.1 ***	543.1 ± 57.5	458.1 ± 57.4 ***
Xc [Ohm]	49.8 ± 5.2 ***	39.1 ± 4.7	29.9 ± 5.8 ***
R [Ohm]	645.8 ± 64.4 ***	541.7 ± 57.4	457.0 ± 57.5 ***
R/H [Ohm/m]	392.3 ± 48.9 ***	324.6 ± 43.4	264.5 ± 39.5 ***
Xc/H [Ohm/m]	30.2 ± 3.4 ***	23.4 ± 3.1	17.3 ± 3.6 ***

**Table 2:** Enriched medication in the dehydrated and hyperhydrated group.

Class	Compound	T	log_2_(FC)		*p*-Value			
			Hyperhydrated	Dehydrated	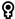		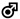	
					Hotelling	B&H	Hotelling	B&H
1	Simvastatin	43	**1.63**	−2.70	0.0178 *	0.0384 *	0.6472	0.6766
	Metoprolol	17	**1.60**	-	0.2066	0.2794	0.0295 *	0.0553
	Candesartan	84	* 1.23	−0.81	0.0180 *	0.0384 *	0.6766	0.6766
	Apixaban	33	**1.16**	−1.45	0.0456 *	0.0805	0.1692	0.2417
	Bisoprolol	131	* 0.84	−0.70	0.0024 **	0.0120 *	0.4214	0.4862
	Amlodipine	61	**0.52**	−0.62	0.1580	0.2370	0.0192 *	0.0384 *
	Amlodipine/Olmesartan	17	-	**1.29**	0.2315	0.2894	0.0067 **	0.0251 *
	Olmesartan	19	-	**0.97**	0.6579	0.6766	0.0067 **	0.0251 *
2	Furosemide	19	** 3.57	-	0.0011 **	0.0082 **	0.0840	0.1400
	Torasemide	93	**1.09**	* −1.69	0.0007 ***	0.0070 **	0.2142	0.2794
3	Allopurinol	46	**1.20**	−0.92	0.0001 ***	0.0015 **	0.3690	0.4428
4/7	Resveratrol	45	−1.87	**1.37**	0.0124 *	0.0384 *	0.0158 *	0.0384 *
5	Mirtazapine	35	−0.97	**0.88**	0.0181 *	0.0384 *	0.1305	0.2061
6	Pantoprazole	85	**1.07**	−1.32	0.0014 **	0.0084 **	0.5671	0.6301
7	Cholecalciferol	220	**0.52**	−0.62	0.0163 *	0.0384 *	0.0001 ***	0.0015 **

Class shows the classification of the compound (1 = Cardiac, 2 = Diuretic, 3 = Endocrinology, 4 = Dementia/Mild Cognitive Impairment, 5 = psychotic/neurological, 6 = gastrointestinal, 7 = nutritional supplements), T = total amount, log2(FC) = binary logarithm, Fisher’s exact test: * = *p* < 0.05, ** = *p* < 0.005; Hotelling’s T test: *p*♀/*p*♂ with and without adjustment for multiple testing using Benjamini & Hochberg’s method, gender-specific *p*-values (referring to extreme group vs. normal hydrated group) and log2(FC) are highlighted in bold if being significant (*p* < 0.05).

## Data Availability

Additional data is available upon request.

## Supplementary Materials

The following are available online at https://www.mdpi.com/article/10.3390/nu13061929/s1, Figure S1: Venn-Diagramm of the overlap between the groups cardiovascular (car, red), renal (ren, yellow), dementia (dem, green) and diuretics (diu, blue), 50 patients remain unclassified (white).
