# Supplementary material for: Association of Drug Application and Hydration Status in Elderly Patients
Source: Nutrients. 2021 Jun 4;13(6):1929. doi: 10.3390/nu13061929 (PMC8226953; doi:10.3390/nu13061929)
Supplement: Supplementary file 1 [file nutrients-13-01929-s001.zip › nutrients-1210365-supplementary.pdf]

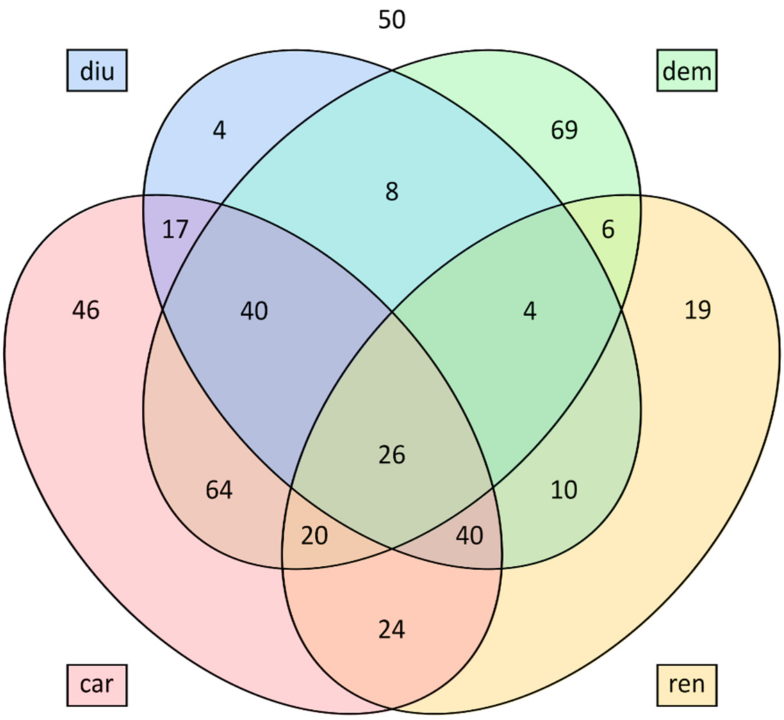

Figure S1 Venn-Diagramm of the overlap between the groups cardiovascular (car, red), renal (ren, yellow), dementia (dem, green) and diuretics (diu, blue), 50 patients remain unclassified (white)
